# Food Processing: The Influence of the Maillard Reaction on Immunogenicity and Allergenicity of Food Proteins

**DOI:** 10.3390/nu9080835

**Published:** 2017-08-04

**Authors:** Malgorzata Teodorowicz, Joost van Neerven, Huub Savelkoul

**Affiliations:** 1 Cell Biology and Immunology Group, Wageningen University & Research, 6708 WD Wageningen, The Netherlands; joost.vanneerven@frieslandcampina.com (J.v.N.); huub.savelkoul@wur.nl (H.S.); 2 Allergy Consortium Wageningen, Wageningen University & Research, 6708 WD Wageningen, The Netherlands; 3 FrieslandCampina, 3818 LE Amersfoort, The Netherlands

**Keywords:** Maillard reaction, advanced glycation end products (AGEs), Maillard reaction products (MRPs), immunogenicity of AGEs, allergenicity of AGEs, Maillard reaction in food

## Abstract

The majority of foods that are consumed in our developed society have been processed. Processing promotes a non-enzymatic reaction between proteins and sugars, the Maillard reaction (MR). Maillard reaction products (MRPs) contribute to the taste, smell and color of many food products, and thus influence consumers’ choices. However, in recent years, MRPs have been linked to the increasing prevalence of diet- and inflammation-related non-communicable diseases including food allergy. Although during the last years a better understanding of immunogenicity of MRPs has been achieved, still only little is known about the structural/chemical characteristics predisposing MRPs to interact with antigen presenting cells (APCs). This report provides a comprehensive review of recent studies on the influence of the Maillard reaction on the immunogenicity and allergenicity of food proteins.

## 1. Food Processing

Most of the food consumed nowadays by developed societies is processed. The diversity of processed food products increased exponentially during the last century, along with the need for more safe, convenient and varied food products. Methods used for food processing can be categorized into two processing types: conventional thermal methods, including pasteurization, sterilization, drying and roasting [4,5] and non-thermal, novel methods such as high pressure treatment [6], electric field treatment [7,8], irradiation [9] or applications of cold plasma [10]. Foods are subjected to thermal processing mainly to preserve them by inactivating microbes (high temperature treatment), to improve their sensory qualities (e.g., flavor, texture, taste, and smell) or to obtain another food product or ingredient from a food source (e.g., protein isolates, cheese, oils). From a biochemical perspective, thermal processing promotes chemical and physical changes of food proteins, and affects protein conformation—and therefore also immunogenicity and allergenicity—by promoting interactions of food proteins with other components present in the food matrix.

## 2. Thermal Processing Induces Conformational Changes in Food Proteins

Native proteins are folded into specific and compact 3D structures. This is determined by primary structure (sequence of amino acids), secondary structure (formation of α-helixes and β-sheets) and tertiary structure. The formation of α-helixes and β-sheets is driven by interactions between polypeptide chains linked together by hydrophobic and hydrophilic interactions, electrostatic interactions and disulfide bonds [11]. All these chemical interactions create a unique protein conformation that is reorganized at all structural levels during heat treatment. Changes in α-helix and β-sheet structures start to occur at heating temperatures above 55 °C, and almost complete loss of secondary and tertiary structure as well as cleavage of disulfide bonds occurs at temperatures above 70–80 °C [12,13]. At the same time, because of protein denaturation, irreversible intermolecular interactions may result in protein aggregation and cross-linking reactions between amino acids, e.g., through formation of lysinoalanine (LAL) [14]. These heat-induced conformational changes of food proteins may further affect digestion and absorption of proteins/peptides by the intestinal epithelium, as well as their recognition by immune cells. Moreover, if sugars are present during the heat treatment, the free amino groups of side chains of amino acids can be blocked due to the Maillard reaction [11,13]. 

## 3. The Maillard Reaction in Food Processing

**Figure 1:**
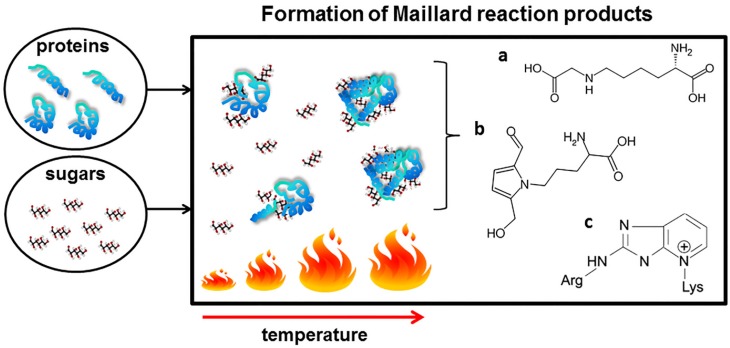
Simplified scheme of the formation of Maillard reaction product during food processing. Amadori rearrangement leads to the formation of a number of advanced glycation end products, among others: (a) Nε-(carboxymethyl)lysine; (b) pyrraline and (c) pentosidine.

One of the best-known interactions between proteins and sugars occurring during heat processing of food is the Maillard reaction (MR), also known as glycation. During the MR, sugars are linked to proteins by a covalent bond between free amino groups of amino acids (mostly lysine and arginine) and the carbonyl groups of a reducing sugar (simplified scheme on Figure 1). 

The MR occurs naturally during regular food processing and meal preparation (cooking, frying, and baking) [15,16,17]. However, the chemistry of the reaction is extremely complex. A cascade of chemical rearrangements including condensation, oxidation and hydration, leads to formation of numerous Maillard reaction products (MRPs). The Schiff bases formed as the first products of the reaction are followed by their Amadori rearrangement and subsequent oxidative modifications (glycoxidations) resulting in the formation of advanced glycation end products (AGEs). The chemical nature of many of the AGEs is unknown due to their heterogeneous and unstable nature. However, a growing number of structurally defined AGEs such as pyrraline, pentosidine and Nε-carboxymethyllysine (CML) have been found in processed food [18,19].

Moreover, the type and amounts of MRPs formed are under the control of factors such as the structural diversity of (poly)saccharides and proteins, reaction temperature and time, ratio of amino group and reducing sugar, pH and water activity [15,20]. MRPs are known to confer functional characteristics to food proteins such as appearance, smell, taste and texture. For this reason, the MR is a relevant reaction for consumers therefore also for the food industry [17,21]. In addition to functional changes of proteins, many studies in the last decade have revealed that the MR also affects biological properties of food proteins such as their digestibility, bioavailability, immunogenicity and consequently their allergenicity. Biochemical and conformational changes of proteins caused by MR may result in masking of existing antibody binding epitopes, but also in creating new structures that are more immunogenic and are thus able to promote the initiation of IgE-mediated allergies [22,23,24,25]. 

## 4. Influence of Maillard Reaction of Digestibility of Proteins

In order to elicit an allergic immune response, food proteins (or peptides thereof) must survive digestion and remain in the gastrointestinal tract for a period of time that is sufficient to induce sensitization. From that perspective, the susceptibility of food proteins to enzymatic hydrolysis seems to be an important factor determining their allergenicity [26,27,28]. Even so, not all food allergens are resistant to digestion [29,30]. As described above, both heating and the MR can alter the susceptibility of proteins to gastrointestinal digestion due to unfolding, heat-induced disulfide bond interchanges, aggregation, the formation of lactulosyllysine, and formation of AGEs, thereby affecting the availability of enzymatic cleavage sites on the protein backbone. 

Heating itself causes unfolding of protein and exposure of linear epitopes and may result in enhanced susceptibility to enzymatic proteolysis as described for β-lactoglobulin heated at the temperature of 90 °C [31]. However, Corzo-Martínez and colleagues demonstrated that denaturation and aggregation of heated β-lactoglobulin caused by MR led to decreased β-LG proteolysis [32]. A band corresponding to intact β-LG was observed on SDS-PAGE picture after trypsin/chymotrypsin digestion of β-LG glycated with galactose and tagatose at 40 °C for one day, even after 1 h of digestion. Resistance to gastrointestinal digestion was more evident in β-LG glycated with galactose than tagatose which was shown previously to be a less efficient sugar in formation of MRPs [33]. Therefore, the results of that study revealed that higher degrees of glycation of bovine β-LG lead to a higher resistance to proteolysis. This could be explained by lower susceptibility of glycated lysine and arginine residues to trypsin/chymotrypsin proteolysis by masking the sites of cleavage [18]. In addition, Maillard reaction-induced protein aggregates may protect proteins during in vitro gastrointestinal digestion, which was shown by inhibition of aggregation of β-LG in the presence of pyridoxamine [32], an effective inhibitor of formation of MRPs on all stages [34]. Those results are in line with a number of other studies that demonstrated impaired enzymatic hydrolysis in vitro due to the MR observed together with decreased protein solubility caused by denaturation, structural rearrangements and aggregation of proteins heated with sugar [25,35,36,37]. In contrast, glycation has also been reported to increase protein solubility [38,39] as well as protein digestibility as it has been shown for lysozyme [40] and codfish parvalbumin [41], suggesting that an effect of MR on protein digestibility may be connected with structural characteristic of the protein that is studied. Moreover, the diversity of conditions used for glycation in the different experiments as pH, time of heating, temperature of heating, ionic strength of the medium, water activity and type of sugar may also explain the disparity often observed in the literature on the influence of MR on protein digestibility. Liu and colleagues showed that the glycation of whey proteins at different conditions of water activity and pH alternates the peptide profiles observed on HPLC chromatograms [42]. The local environment of lysine changes in differently unfolded proteins at pH 5, 7 or 9, which affected the susceptibility for glycation, the type of formed MRPs (e.g., formation of agglomerates), and subsequently modified the protease action, resulting in a different peptide composition after enzymatic hydrolysis [42].

The number of studies involving in vivo experiments investigating an effect of MR on digestibility of food proteins is limited to date. A recently published study performed on humans compared the effects of diets with different MRP contents on dietary protein utilization in adolescent males. The study revealed that a diet high in the MRPs limits the digestibility of proteins since 47% higher fecal nitrogen excretion, 12% lower apparent nitrogen absorption and 6% lower nitrogen digestibility was observed in the group consuming diet high in MRPs [43]. Hellwig and colleagues [44] have shown that the human colonic microbiota are able to degrade the following MRPs: Nε-fructosyllysine, CML and pyrraline. This suggests that the released glycated peptides and amino acids may be used by microbes as the source of energy or can be transported via intestinal barrier as it was proposed for small pyrraline peptides [45]. This shows the relevance of microbiota in the degradation process of Maillard reaction-modified proteins and therefore also their absorption in the intestine. 

## 5. Absorption of MRPs in Intestine

The question if the glycated protein/peptide can have immunological effect in vivo depends on whether these molecules are available in the gastrointestinal mucosa to be absorbed into the circulation and subsequently to get in contact with the immune system. Dietary MPRs were shown to appear in the circulation and/or urine of human subjects after consumption of MRPs rich diet [18,46,47,48,49,50,51,52]. The study performed by Hellwig and colleagues on glycated casein samples revealed that fructoselysine and (CML) are released after digestion bound to peptides smaller than 1000 Da, which makes them available for absorption [52]. This was confirmed in the studies performed in the rat model showing that advanced MRP such as CML, pyrraline and pentosidine may be absorbed by the gut. Ingested dietary CML in rats appeared to be approximately 26.0–29.0% excreted in the urine, and 15.0–22.0% excreted in feces [14,53]. Approximately 1.7% of dietary CML accumulated in the circulation, kidney and liver and approximately 50.0% of the ingested CML was not recovered. This was later confirmed in a human study where 31.2% of ingested dietary CML was excreted in the feces, 14.4% in the urine, and 54.4% left unrecovered [48]. More than 60% of dietary pyrraline and approximately 2.0% of dietary pentosidine were excreted in urine in humans [49,50]. Different percentage of recovery of total diet amount of AGEs found in urine suggest different resorption and metabolic pathways of individual Maillard products [50]. In addition, LAL, a compound formed by cross-linking of protein, was found in the urine, plasma, liver and kidneys of rats fed with the diet with low and high LAL-content [14] although it has been reported to be released during digestion process into larger peptides of at least 30–40 amino acids [52]. 

Cross-linking of proteins seems to reduce an epithelial uptake of proteins although promote an uptake through Peyer’s patches as was shown for crossed-linked β-lactoglobulin and α-lactalbumin [54,55]. In addition, the larger agglomerates, as well as other MRPs can be further metabolised by intestinal microbes [44,56,57] resulting in formation of new bioactive compounds but also modulating the intestinal microbiota composition in humans [58,59]. The fact that the dietary protein-bond AGEs, such as CML, pentosidine and pyrraline, are available in the gastrointestinal tract and circulation means that they can also interact with the immune cells. 

## 6. Influence of Maillard Reaction on Immunogenicity of Proteins

### 6.1. MR-Modified Proteins and Allergic Sensitization

Even though the role of AGEs in chronic inflammatory diseases is more well-known and studied [47,60,61], evidence is emerging that AGEs also play a role in allergy. As this paper focuses on allergy, the role of AGEs in chronic inflammation will not be discussed in details.

Allergy is commonly divided in two phases. An initial phase of allergic sensitization represents principally a particular immune reaction leading to the formation of allergen-specific IgE antibodies. IgE antibodies can crosslink adjacent cell-bound IgE molecules on basophils and mast cells upon repeated exposure to allergens, leading to degranulation and the release of mediators, including histamine, prostaglandins, leukotriens and tromboxanes. These mediators cause the typical symptoms of a type 1 (within 20 min) IgE-mediated allergy, including rhinitis, atopic dermatitis, allergic asthma, and occasionally even anaphylaxis [62].

**Figure 2:**
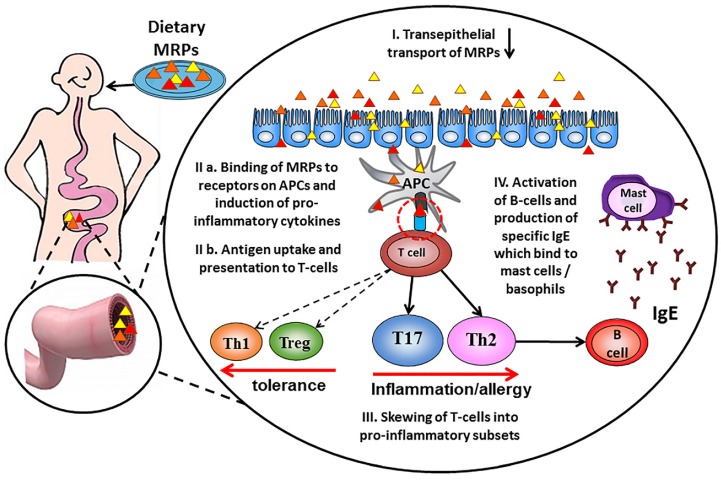
Schematic contribution of dietary Advanced glycation end products in the allergic sensitization process. Dietary Maillard reaction products (MRP) are taken up in the gut by crossing the epithelial barrier (I), leading to antigen uptake by mucosal dendritic cells and presentation of peptides to specific T-cells (II). Activated antigen-specific helper Th cells differentiate into pro-inflammatory and allergy-inducing Th17 and Th2 subsets (III). Allergen-specific B-cells become activated upon ligand binding and start the production of allergen-specific IgE antibodies (IV) that bind to mast cells and basophils and become detectable in the circulation.

Immunogenicity of proteins is thus dependent on their ability to eventually induce adaptive T-and B-lymphocyte responses during the allergic sensitization phase (see Figure 2). This is strongly influenced by the efficiency of antigen uptake and processing, as well as by the activation status and production of cytokines by myeloid antigen presenting cells (APC: monocytes, macrophages, and dendritic cells). Receptor-mediated endocytosis—as opposed to pinocytosis—is an efficient way of antigen uptake that facilitates adaptive immune responses at low antigen exposure and also induces activation and cytokine production by antigen presenting cells. APCs such as dendritic cells (DC) can use Fc receptors [63], RAGE (receptor for advanced glycation end products) [64,65], dectin-1, 2 and 3 [66,67], DC-SIGN [68], galectin-3 [69] and mannose receptors [70] for efficient antigen uptake. For example, it is known that IgG-immune complexes are more immunogenic because they are targeted to CD32/FcɣRII receptors on APCs [71]. This was also shown for an uptake of allergen-IgE complexes via CD23/FcεRII, resulting in efficient T cell activation at 100-fold lower antigen concentrations [72]. These findings suggest that interaction with specific receptors on APC may be of importance to understand an immunogenic character of dietary AGEs (see Figure 2).

### 6.2. Interaction of MR-Modified Proteins with Receptors Present on APCs

To induce an allergic immune response the MR-modified protein need to be recognized and taken up by antigen presenting cells (APCs) and subsequently presented to T-cells [24]. Recently more evidence has been found that some MRPs may function as activators of dendritic cells (DCs) via targeting AGE receptors [23,73,74,75]. Several receptors mediating antigen uptake, activation and maturation in DCs were identified as potential receptors for dietary MRPs, including AGE-receptor complex (AGE-R1/OST-48, AGE-R2/80K-H, AGE-R3/galectin-3) [76,77,78], members of the scavenger receptor family A (SR-A) and B (SR-B) [23,24,75,79,80,81] as well as mannose receptor [82,83,84]. Dry roasted Ara h1 was bound to the scavenger receptor, CD36 and the receptor for advanced glycation end products (RAGE) [24,85]. 

RAGE is the most studied receptor that can recognize and bind dietary AGEs [24,42,85,86,87,88]. Cellular signaling due to AGE–RAGE interactions seem to be a key component in pro-oxidative and pro-inflammatory condition [89] and this may be involved in enhanced allergic sensitization [85]. Soluble RAGE (sRAGE), the extracellular ligand-binding domains of RAGE present in the circulation, competes with membrane RAGE in binding AGEs acting as a decoy domain receptor [90,91]. Binding of AGEs to sRAGE, in contrast to interactions with membrane form of RAGE, does not result in inflammatory signal transduction therefore sRAGE acts as inhibitor of RAGE-AGE signaling and is potentially applicable for the treatment of various AGE-related diseases including diabetic cardiovascular complications [92,93], diabetic kidney disease [94] and a number of aging-related diseases including atherosclerosis, cataracts, Alzheimer’s disease and Parkinson’s disease [95,96]. 

The recent study of Liu and colleagues revealed that whey proteins glycated by dry heating (at 130 °C) interacts with sRAGE. The strength of binding was positively correlated with the time of heating and the formation of agglomerates and was more prominent in the samples with lower water activity [86]. These findings are in line with other studies showing binding of AGE-modified peanut allergens to recombinant form of RAGE [24]. The study performed by Zill and colleagues [85,97] revealed RAGE-mediated activation of both Caco-2 cells and RAGE-transfected HEK-293 cells by chemically defined food-derived products, both, AGEs and non-AGEs. This suggests that diet-derived AGEs may activate the antigen presenting cells via RAGE. The study performed by Hou and colleagues suggest a positive correlation between the level of AGEs accumulated in the circulation of patients with chronic kidney disease and the expression of RAGE on monocytes isolated from these patients [98]. The enhanced expression of RAGE was strongly correlated with plasma levels of pentosidine, plasma levels of tumor necrosis factor alpha (TNF-α), monocyte activation markers, and the systemic acute phase reactant, C-reactive protein [98]. Hilmenyuk and colleagues demonstrated activation of RAGE on immature DC via interaction with AGE-modified ovalbumin (OVA). Increased expression of RAGE on immature DCs exposed to AGE-modified OVA was seen as well as enhanced activation of the transcription factor NF-κB compared to DCs exposed to non-modified OVA [83]. The ligation of RAGE by CML was shown to upregulate the RAGE expression in human neuroblastoma cell line SH-SY5Y [87] as well as enhance the expression of vascular cell adhesion molecule-1 (VCAM-1) on endothelial cells [99]. These data suggest that AGE-RAGE interaction may result in NF-κB activation as well as upregulation of RAGE expression on immune cells what in consequence may result in secretion of pro-inflammatory cytokines and thus activation of APCs. In opposite to these results other studies show that CML-modified proteins [100] as well as Maillard-reaction modified β-lactoglobulin [101] and coffee [102] are not able to stimulate inflammatory signaling pathways in RAGE-expressing human cell lines [100,101]. Interestingly, ovalbumin modified by pyrraline, the other AGE, was also not shown to interact with RAGE [23]. Thus, the discussion on the ligation of RAGE with food-derived AGEs and its physiological consequences remains open and more data on activation of RAGE by AGEs are needed to prove the role of RAGE in the activation of APCs during the allergic sensitization process. 

### 6.3. Influence of MR-Modified Proteins on T-Cell Activation and Polarization

Interaction of AGEs with receptors present on APCs may result in internalization and therefore presentation of antigen to T-cells. Ilchman and colleagues demonstrated that AGE-modified OVA was taken up much more efficiently by bone marrow-derived murine myeloid dendritic cells (mDCs) than native OVA, and enhanced activation of OVA-specific CD4+ T cells [75]. Scavenger receptor class A type I and II (SR-AI/II) were identified as receptors mediating the uptake of AGE-OVA [75]. These results are in line with a study using human DCs as a model for an uptake of FITC-labeled AGE-modified OVA. Enhanced uptake of AGE-modified OVA was mediated by mannose receptor, scavenger receptor and macropinocytosis. Co-culturing of CD4+ T cells with AGE-OVA-loaded mature DCs induced greater Th2 cytokine production (IL-5, IL-4, and IL-6), while OVA-loaded DCs induced a significant Th1 or regulatory cytokine profile [83]. The study of Moghaddam and colleagues performed on dry roasted peanut proteins confirm the ability of dietary AGEs to target antigen presenting cells via RAGE and CD36. Moreover, mice sensitized with dry-roasted peanut extract showed higher IL-4, IL-5 and IL-13 secretion by mesenteric lymph node cells showing skewing of T cell response into Th2 when compared with mice sensitized with non-treated peanut extract. High reactivity of mice primed with dry roasted peanut to raw peanut antigens suggest that roasting enhances immunogenicity of peanut extract having an important impact on the priming step of sensitization [24]. 

Heilmann and colleagues aimed to identify glycation structures enhancing T-cell immunogenicity of a food allergen by modification of OVA with different AGEs such as CML, CEL and pyrraline. To assess the T-cell immunogenicity of glycated OVAs, murine OVA-specific CD4+ T-cells were co-cultured with bone marrow-derived DCs in the presence of differently processed OVA samples. Pyrraline modified OVA enhanced CD4+ T-cell immunogenicity, as evidenced by increased IL-2 production, higher production of IFN-γ and IL-17A when compared with native OVA. Moreover, pyrraline-OVA and AGE-OVA were efficiently taken up by BMDCs via SR-A. In addition to antigen uptake, cell maturation is required for DCs to gain their full T-cell stimulatory capacity. However, no differences in expression of co-stimulatory molecules CD40, CD80, CD86, and MHC class II on the cell surface of DC were seen suggesting that pyrraline modification does not induce BMDC maturation. This is in line with observations of Moghaddam and colleagues [24], who suggested enhanced targeting and presentation via AGE receptors rather than conventional DC maturation may be implicated in the increased immunogenicity of DR peanut antigens in vivo. However, this is in contrast with the work of Buttari and colleagues who showed that AGEs of plasma β2 glycoprotein I (β2 GPI) triggered the maturation of monocyte-derived human DCs and polarized allogenic naive CD4+ T-cells into Th2 cells in a co-culture with matured DCs [73]. These different observations could be explained by heterogeneity of the structures MRPs and the expression profiles of receptors in these human and murine DCs. 

### 6.4. Role of Agglomeration in Immunogenicity of MR-Modified Proteins

These results raise the question which structural changes triggered by MRPs can explain the immunogenicity of AGEs and capabilities to initiate the polarization of allogenic naive CD4+ T-cells into Th2 cells. Maillard reaction caused agglomeration of proteins was excluded as a major contributor to increased immunogenicity of proteins in the studies of Moghaddam and colleagues [24] and Heilmann and colleagues [23] since the samples underwent multiple rounds of filtration and centrifugation that depleted cross-linked species. However, the agglomeration of MR-modified proteins and level of cross-linking were not measured in these studies. Moreover, food processing of proteins promotes the formation of β-sheet-rich, fibrillar structures [103] known to possess high affinity binding to RAGE [104] and CD36 [105]. Pasteurization caused aggregation of beta-lactoglobulin and alpha-lactalbumin enhanced uptake of cross-linked proteins via Peyer’s patches, which promoted significantly higher Th2-associated antibody and cytokine production in mice than their native counterparts [54,55]. These results suggest that not only Maillard reaction but also protein cross-linking can enhance immunogenicity of proteins and their sensitizing capacity.

The research outcomes discussed above provide strong evidence that some dietary AGEs can bind to receptors on antigen presenting cells and thus modify T-cell immunogenicity (the schematic contribution of dietary AGEs in the allergic sensitization process is presented on Figure 2). However, information about selectivity of AGEs structures binding to AGEs receptors is very limited. The heterogeneity of AGEs and the diversity of their receptors indicate that more study is required to elucidate the precise receptors and pathways implicated in enhanced DC-mediated uptake and presentation of antigens to T cells. 

### 6.5. Influence of MR-Modified Proteins on B-Cells Switching and the Production of Antigen Specific IgG and IgE

When membrane-bound immunoglobulin (Ig) of naïve B cells come in contact with specific dietary antigens, and are activated by ligation of the surface molecule CD40 to CD40L on activated Th2 cells that produce IL-4 and IL-13 , the B cells are induced to switch to produce IgE. Naïve B cells further differentiate and proliferate into activated plasma cells synthesizing and secreting antigen-specific IgE (see Box 2). Lymphocytes activated in the GALT leave through the draining lymphatics and reach the MLN, where they stay for a period for further differentiation, before migration into the bloodstream [62,106]. Moghaddam and colleagues demonstrated that BALB/c mice primed subcutaneously with soluble fractions of peanut protein extract from raw or dry roasted (DR) peanuts show enhanced peanut-specific IgG titers in DR-primed groups. These results were confirmed in intra-gastric gavages of DR and raw peanut extracts showing 100-fold higher IgG titers, enhanced titers of anti-peanut IgE as well as functional basophil degranulation in DR group. Moreover, mesenteric lymph node cells from DR but not raw peanut protein-primed mice proliferated robustly in response to raw and DR peanut extract with the dominance of IL-4 and IL-5 over IFN-γ and TNF-α. The authors suggested that the observed increased immunogenicity of DR peanut antigens can be explained by selective targeting, activation and presentation of antigen via binding to AGE receptors on DCs [24].

## 7. Influence of Maillard Reaction on Recognition of Food Allergens by Specific IgE

**Figure 3:**
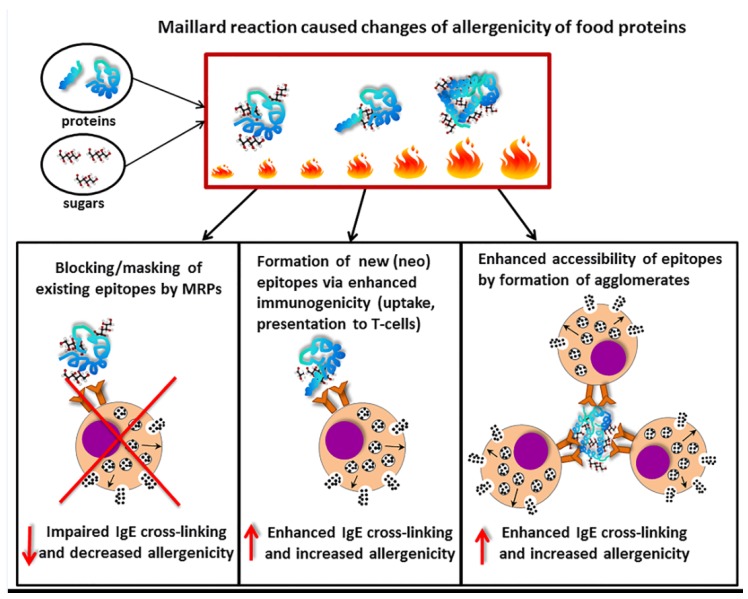
Modulation of allergenicity of food proteins by the Maillard reaction. Upon food processing using heating, reducing sugars will react with primary amino groups on amino acids from allergen proteins to result in Maillard reaction products (MRP). Consequently, these MRP may block epitopes thereby preventing IgE binding and crosslinking, and subsequent mediator release. This results in reduced allergenicity of the altered food allergens. Alternatively, MRP may lead to the exposure of neo-epitopes leading to enhanced uptake by antigen-presenting cells and exposure to specific T-cells and this enhanced possibility for IgE cross-linking may lead to enhanced allergenicity. Lastly, MRP may lead to the formation of agglomerates of allergen molecules resulting into enhanced IgE cross-linking and increased allergenicity. The ratio between these several possibilities determines the final outcome of the mediator release capacity of the MRP altered food allergens.

MR induced during food processing may also modulate binding potential of specific IgE to food allergens. This can be induced by: (a) disruption of the conformational and linear epitopes accompanied with the changes of the tertiary and secondary structure that impair the IgE binding potential of the protein [12,106,107]; (b) formation of agglomerates carrying high number of epitopes that cause enhanced degranulation capacity of basophils [108]; or (c) formation of new epitopes due to aggregation and/or Maillard reaction [24] (Figure 3). These new epitopes called neo-allergens are able to target APCs resulting in antigen presentation and subsequently modulating T-cell differentiation as well as a production of antigen-specific IgE. Production of specific IgE is therefore a consequence of sensitization phase by interaction of immunogenic MRPs with APCs (see Box 1 and Box 2). Moghaddam and colleagues observed that enhanced anti-raw peanut IgE titers in mice sensitized with dried peanut extract versus those sensitized with raw peanut extract was also reflected in enhanced degranulation of basophils [24]. 

The MR was shown to either reduce or enhance IgG and/or IgE binding capacities of some food allergens [25,109,110,111,112,113]. For instance, the proteins that belong to pathogenesis-related (PR) protein family and being the homologous to birch pollen allergen Bet v 1 show reduced IgE binding capacity after processing with sugar as it was shown for Pru av 1 [109] and Cor a 1 [114]. This can be explained by a masking effect of carbohydrates reducing an accessibility of epitopes for IgE binding (Figure 3). In contrast to (PR) protein family MR was shown to enhance IgE binding capacity to peanut proteins and scallop tropomyosin [115,116,117]. Thus, the influence of MR on IgE binding seems to depend on physicochemical properties of proteins (hydrophobicity, size, amino acid composition, charge) as well as on conditions of MR (type of sugar, time, water activity, pH, temperature, presence of salts) [118,119].

The study of Vissers and colleagues showed reduced allergenicity of MR-modified peanut allergen Ara h 1 in IgE binding test while enhanced β-hexosaminidase release from basophils upon incubation with the same MR-modified allergen [113]. The authors suggest that MR-induced agglomeration of Ara h 1 may be responsible for the observed increased capacity of antigen to cross-link the IgE and initiate the mediator release from RBL-2H3 cells (see Box 1). That indicates a need to include the functional assays, next to the IgE binding tests, in the studies on allergenicity of MRPs to be able to link the results to development of acute complaints of a clinically observable allergic response (see Box 3). Based on the functional basophil activation test (BAT) Cucu et al. showed that two out of six hazelnut allergic patients showed enhanced basophil activation after exposition to MR-modified hazelnut extract [114]. Other study revealed that 70% of the patients (out of 15) sensitized to soy showed enhanced basophil activation in BAT assay upon incubation of basophils with MR-modified soy proteins when compared with soy proteins modified only by heating (without sugars) [22]. Moreover study of Vojdani and colleagues revealed increased levels (3–8-fold) of specific IgE against processed food antigens in 31% of the patients when compared to raw food antigens [120]. These results suggest that some of the patients are sensitized against processed food rather than raw and the MR may play a crucial role in the enhancing of immunogenic potential of food allergens as it was already showed by Moghaddam and colleagues [24]. Therefore, the diagnosis of food allergy could be improved by incorporation of processed and MR-modified food allergens next to the raw ones into the diagnostic tests.

Box 1 **Immunochemical properties of allergens.**

Allergens are generally proteins that, when exposing genetically susceptible individuals frequently and in low doses (<1 µg) on a mucosal surface are able to induce a Th2 response associated with the production of IL-4, IL-5, and IL-13 leading to the synthesis of allergen-specific IgE antibodies.

At present, no identified antibody characteristics and no identified structural features of IgE binding epitopes seem to be associated with the phenotype of the food allergic disease. Potential allergens must necessarily contain B-cell epitopes to which IgE can bind, and T-cell epitopes capable of inducing a Th2 type response. Peptide sequences of T-cell epitopes (10–15 amino acid residues long) show no general homology across allergen families and thus it has been proven impossible to identify a consensus sequence for an allergenic epitope [121].

IgE binding allergen epitopes generally comprise conformational and more hydrophobic patches present on the allergen. A significant proportion of the IgE is directed against the glycosylated B-cell epitopes [122]. This might be a consequence of the hundred-fold increased cellular uptake of glycosylated proteins and peptides by antigen-presenting cells compared to their non-glycoslyated counterparts and resulting in enhanced immune responses [123]. However, others suggest that glycosylation is not a common critical determinant of allergenicity as food allergens comprise both glycoproteins as well as non-glycosylated proteins [124].

An epitope is identified by its ability to bind antibodies and is suggested to consist of a recognizable sequence of 6–15 amino acids (covering a 11–13 nm distance) contributing to the binding between epitope and antibody molecule [125]. Two of these identical epitopes need to span a distance between 8 and 24 nm, with a single amino acid being in the order of about 0.5 nm [126,127]. A linear unit potentially leading to degranulation would then comprise a distance of 20–54 nm equaling 40–108 amino acids (4.400 to 11.880 Da) [128]. In a 20 kDa allergen this would represent 2–5 units, while a 200 kDa allergen would harbor 16–45 units. Indeed, most allergens have only one to five immunodominant epitopes. A 40 amino acid unit would be sufficient to induce IgE antibody formation and combine the potential to result in degranulation upon binding to IgE on sensitized mast cells.

Box 2 **Immunochemical properties of IgE.**

IgE molecules have a 12% carbohydrate content with oligosaccharides asparagine-linked at six places all in the first three of the four constant region domains, they have a limited segmental flexibility [129]. Therefore, IgE molecules are generally considered to be more rigid than IgG molecules putting emphasis on the importance of the inter-epitope distance resulting in proper IgE binding. This way a binding stoichiometry of two can be reached as closely as possible resulting in a high binding affinity of the allergen-specific IgE antibodies [130]. The relevance of IgE affinity is illustrated in studies showing that in allergic individuals, the peanut allergen Ara h 2-specific IgE affinity correlated with the severity of the allergic disease, but not with the level of specific IgE [129,131]. In allergic individuals, IgE concentrations in the circulation may reach over 10 times the normal level (≈150 ng/mL), and who have an increased risk of developing allergies. However, the concentration of IgE in the serum of healthy individuals is 10^4^ times less than that of IgG.

These IgE antibodies have the capacity to bind to IgE Fc epsilon receptors with high affinity (K_a_ ≈ 10^10^ M^−1^). This exceptionally high affinity is mainly a reflection of the very slow dissociation rate with a half-life of about 20 h for circulating IgE. The residence time on mast cells in tissues is further extended to more than 14 days by restricted diffusion and rebinding to cell receptors [130].

Box 3 **Advantage of functional tests on IgE binding assays in the diagnosis of food allergy.**

Diagnosis of allergy is routinely based on binding of IgE antibodies to the relevant allergen in a serological test. Principally, this binding of one Fab fragment to a single epitope results in a positive reading representing immunogenicity. This mostly conformational monovalent binding interaction is not related to the capacity to induce an allergic response resulting into mediator release. For this reaction it is essential to achieve cross-linking of several specific IgE molecules bound to adjacent FcεRI on mast cells and basophils (hence allergenicity). Principally, this elicitation phase requires a multivalent interaction between (mostly linear) allergen multi-epitopes and multiple Fab fragments on IgE antibodies. This allergen capacity can be analyzed ex vivo with blood-derived basophils or in vitro by using e.g., the rat basophilic leukemia (RBL)-HE3 cell line and is generally called allergenicity of the allergen. To analyze the basophil degranulation capacity sensitive assays are required as these cells comprise only 0–2% of leukocytes equaling 1–8 × 10^4^ cells/mL [130]. This different capacity of allergens should be taken into account when relating the functional activity of allergens to the clinical symptoms of allergy. High affinity Fcε receptors are abundantly expressed (6000–600,000 receptors per cell) on mast cells in tissues and basophils in the blood. The number of FcεRI per basophil varies between different donors (range 29,000–680,000) and is also related to the IgE concentration in the serum [131]. Upon IgE binding to their specific receptors, these cells are sensitized and therefore the individual is called sensitized. Minimally, 2000 of these FcεRI bound specific IgE molecules need to be cross-linked by the relevant epitopes on the allergen in order to cause degranulation of the mast cell and/or basophil resulting in the release of the mediators and the development of acute complaints of a clinically observable allergic response. Therefore, functional assays such as histamine, β-hexosaminidase release assays or basophil activation tests should be included next to the conventional IgE binding tests to study the influence of Maillard reaction on allergenicity of proteins.

## 8. Conclusions

In conclusion, the MR can alter immunoreactivity towards food proteins, and MR-modified proteins may enhance the immune response by selective interaction with APCs carrying receptors for AGE. In addition, it has become clear that the presentation of MR-modified allergens to T-cells may skew the subsequent T-cell differentiation into Th2 cells producing IL-4, IL-5 and IL-13, which are responsible for the initiation of IgE antibody production. Some studies also observed enhanced mediator release from basophils incubated with MR-modified proteins that may be resulting from: (a) the immunogenic potential of MRPs enhancing the sensitization; and/or (b) agglomeration leading to more efficient cross-linking and therefore mediator release. These findings reveal a need for better understanding of the influence of MR on both the sensitization phase as well as the development of the symptoms of the allergy. Understanding the mechanisms involved in immunoreactivity of AGEs would help to improve the diagnostics of food allergy as well as develop optimized conditions for food processing to control the rate of MR.
